# Uracil restores susceptibility of methicillin-resistant *Staphylococcus aureus* to aminoglycosides through metabolic reprogramming

**DOI:** 10.3389/fphar.2023.1133685

**Published:** 2023-01-24

**Authors:** Lvyuan Fan, Zhiyu Pan, Xu Liao, Yilin Zhong, Juan Guo, Rui Pang, Xinhai Chen, Guozhu Ye, Yubin Su

**Affiliations:** ^1^ MOE Key Laboratory of Tumor Molecular Biology, Guangdong Provincial Key Laboratory of Bioengineering Medicine, Department of Cell Biology and Institute of Biomedicine, National Engineering Research Center of Genetic Medicine, College of Life Science and Technology, Jinan University, Guangzhou, China; ^2^ Center for Excellence in Regional Atmospheric Environment, and Key Laboratory of Urban Environment and Health, Institute of Urban Environment, Chinese Academy of Sciences, Xiamen, China; ^3^ Guangdong Provincial Key Laboratory of Microbial Safety and Health, State Key Laboratory of Applied Microbiology Southern China, Guangdong Institute of Microbiology, Guangdong Academy of Sciences, Guangzhou, China; ^4^ Institute of Infectious Diseases Shenzhen Bay Laboratory, Shenzhen, China

**Keywords:** methicillin-resistant *Staphylococcus aureus*, uracil, gentamicin, TCA cycle, ROS, metabolic reprogramming

## Abstract

**Background:** Methicillin-resistant *Staphylococcus aureus* (MRSA) has now become a major nosocomial pathogen bacteria and resistant to many antibiotics. Therefore, Development of novel approaches to combat the disease is especially important. The present study aimed to provide a novel approach involving the use of nucleotide-mediated metabolic reprogramming to tackle intractable methicillin-resistant *S. aureus* (MRSA) infections.

**Objective:** This study aims to explore the bacterial effects and mechanism of uracil and gentamicin in *S. aureus.*

**Methods:** Antibiotic bactericidal assays was used to determine the synergistic bactericidal effect of uracil and gentamicin. How did uracil regulate bacterial metabolism including the tricarboxylic acid (TCA) cycle by GC-MS-based metabolomics. Next, genes and activity of key enzymes in the TCA cycle, PMF, and intracellular aminoglycosides were measured. Finally, bacterial respiration, reactive oxygen species (ROS), and ATP levels were also assayed in this study.

**Results:** In the present study, we found that uracil could synergize with aminoglycosides to kill MRSA (USA300) by 400-fold. Reprogramming metabolomics displayed uracil reprogrammed bacterial metabolism, especially enhanced the TCA cycle to elevate NADH production and proton motive force, thereby promoting the uptake of antibiotics. Furthermore, uracil increased cellular respiration and ATP production, resulting the generation of ROS. Thus, the combined activity of uracil and antibiotics induced bacterial death. Inhibition of the TCA cycle or ROS production could attenuate bactericidal efficiency. Moreover, uracil exhibited bactericidal activity in cooperation with aminoglycosides against other pathogenic bacteria. In a mouse mode of MRSA infection, the combination of gentamicin and uracil increased the survival rate of infected mice.

**Conclusion:** Our results suggest that uracil enhances the activity of bactericidal antibiotics to kill Gram-positive bacteria by modulating bacterial metabolism.

## Introduction

Methicillin-resistant *Staphylococcus aureus* (MRSA) is a clinically relevant highly virulent pathogen, responsible for a substantial number of hospital and community infections. Widespread use of antibiotics, resulting in evolution of drug resistance genes and antibiotic insensitivity in MRSA (Grumann et al., 2014; Lee et al., 2018; Kanth et al., 2021). Upon infection, MRSA produces numerous virulence factors including hemolysis, which upon entering the bloodstream can potentially result in sepsis and multiple organ dysfunction syndrome (Grumann et al., 2014). Currently, antibiotics remain the primary choice for treatment of bacterial infection. However, most common antibiotics, such as aminoglycosides, penicillin, macrolides, tetracyclines, fluoroquinolones, sulfonamides, and rifampin are quite ineffective against MRSA (Blázquez et al., 2014; Khosravi et al., 2017; Takada et al., 2017). Therefore, there is an urgent need to develop new approaches to restore the efficacy of antibiotics in controlling antibiotic-resistant pathogens such as MRSA.

Many reports have indicated that the metabolic state of bacteria mediated antibiotic resistance (Peng et al., 2015b; Stokes et al., 2019; Lopatkin et al., 2021; Kok et al., 2022). Reprogramming metabolomics approach by modulating crucial metabolites can restore bacterial antibiotic sensitivity and elevate antibiotic efficacy (Peng et al., 2015a; Kuang et al., 2022). For example, antibiotic-resistant metabolome was reprogrammed to antibiotic-sensitive metabolome using exogenous agents such as alanine, glucose, fructose, and glutamic acid (Peng et al., 2015b; Su et al., 2018; Tang et al., 2021). These metabolites mainly regulate central carbon and energy metabolism, thereby increasing bacterial susceptibility to antibiotics. Additionally, reactive oxygen species (ROS) have previously been associated with antibiotic resistance (Memar et al., 2018). Specifically, reduction in ROS production has been attributed to decreased central carbon metabolism in bacteria with gentamicin resistance (Zhang et al., 2019). Glucose reprogramming activates central carbon metabolism and promotes ROS production, thereby improving the bactericidal effect on antibiotic-resistant *Vibrio alginolyticus* (Zhang et al., 2020). Further analysis showed that most resistant bacteria exhibited reduced central carbon metabolism and energy metabolism in response to different antibiotics (Gardner et al., 2018; Liu et al., 2019; Su et al., 2020). Therefore, reprogramming central carbon and energy metabolism using key metabolites provides a promising strategy to enhance antibiotic efficacy (Peng et al., 2015b; Su et al., 2018; Li et al., 2020).

Metabolic reprogramming strategies have been developed in an attempt to use metabolites and compounds to enhance the activity of aminoglycosides, penicillins, fluoroquinolones, and tetracyclines against antibiotic-resistant bacteria (Marques et al., 2014; Peng et al., 2015b; Meylan et al., 2017; Liu et al., 2020a; Jiang et al., 2021; Zhao et al., 2021). Aminoglycosides are widely used to treat various clinical bacterial infections and are regarded as highly effective antibiotics. The bactericidal activity of aminoglycosides involves its synergistic effects on cellular respiration and proton motive force (PMF) (Lobritz et al., 2015; Wang et al., 2022). Enhancing PMF-dependent uptake of aminoglycosides further stimulates killing of antibiotic-resistant bacteria (Allison et al., 2011; Peng et al., 2015b; Crabbe et al., 2019). In particular, the compound n-butanol was recently reported to potentiate the inhibitory effect of aminoglycosides against MRSA by elevating antibiotic uptake (Lv et al., 2022). Therefore, with a growing number of studies broadening the scope of metabolic interventions, more metabolites or compounds have been identified to augment the activity of existing antibiotics against drug-resistant bacteria.

Nucleotides are important bioactive metabolites that play key roles in various biochemical processes. Nucleotide metabolism is directly linked to cellular homeostasis, and is involved in carbohydrate metabolism, oxidative phosphorylation, and nucleotide biosynthesis (Roy et al., 2016). Moreover, recent studies have demonstrated a role for nucleotide metabolism in regulating antibiotic efficacy against bacterial pathogens (Lopatkin and Yang, 2021). For example, an integrated “white-box” biochemical screening and machine learning approach revealed that nucleotides associated with purine biosynthesis improve the bactericidal effect of antibiotics against *Escherichia coli* (Yang et al., 2019). Similarly, inosine promotes antibiotic-mediated killing of multidrug-resistant uropathogenic bacteria, while thymine potentiates the activity of antibiotics against various Gram-negative bacteria (Liu Y. et al., 2021; Zhao et al., 2021). However, whether nucleotides regulate the susceptibility of antibiotic-resistant Gram-positive bacteria to antibiotics remains unclear. Furthermore, the mechanisms underlying nucleotide-induced metabolic reprogramming of bacteria are still unexplored.

In this study, we found that exogenous uracil synergizes with aminoglycosides to kill Gram-positive pathogenic bacteria. Subsequently, a GC-MS-based metabolomics approach revealed that uracil activates the TCA cycle, and increases ROS generation, thereby improving the efficacy of existing antibiotics *in vitro* and *in vivo*.

## Materials and methods

### Bacterial strains, culture conditions, and chemicals

Methicillin-resistant *S. aureus* USA300_FPR3757 was kindly provided by Hua Zhou, from Zhejiang University. MRSA252, *Corynebacterium diphtheriae*. *S. aureus* Newman, and *Streptococcus agalactiae* were from the collection of our Lab. Strains were identified by 16S rRNA sequencing. USA300, MRSA252, *C. diphtheriae* and Newman were grown at 37°C for 12–16 h in 30 mL Luria-Bertani (LB) broth (HuanKai Microbiology Technology Co., Ltd., Guangdong, China) with 220 rpm shaking. *S. agalactiae* was grown at 30°C for 12–16 h in 30 mL of LB broth with 220 rpm shaking. Uracil, thymine, cytosine, adenine, guanine, malonic acid, and antibiotics were purchased from Aladdin (Aladdin Biotech Co., Ltd., Shanghai, China). N-Acetyl-L-cysteine (NAC) was purchased from Solarbio (Beijing Solarbio Science and Technology Co., Ltd., Beijing, China). Citric acid, fumaric acid, succinic acid, and malic acid were purchased from Macklin (Macklin Biotech Co., Ltd., Shanghai, China). Carbonyl cyanide 3-chlorophenylhydrazone (CCCP) and 2′,7′-dichlorofluorescein diacetate (DCFH-DA) were obtained from Sigma-Aldrich (USA). 3,3′-diethyloxacarbocyanine iodide (DiOC23)) was purchased from Invitrogen (USA).

### Measurement of minimum inhibitory concentration (MIC)

The minimum inhibitory concentrations of gentamicin against USA300 and *E. coli*-R_Gen_ were determined as previously described (Wiegand et al., 2008). Briefly, cells were cultured in 96-well plates containing LB medium with doubling serially diluted gentamicin ranging from 0.01 to 160 μg/mL. Overnight bacterial cultures were diluted 1:100 in fresh LB medium and cultured till OD_600_ reached 0.5. The tray contained 90 µL of LB medium, with a series of two-fold dilutions of gentamicin and 10 µL of logarithmic-phase cells at 5 × 10^6^ CFU/mL. After overnight incubation, the minimal antibiotic concentrations exhibiting no visible growth was recorded as the MIC value. Three biological replicates were performed.

### Antibiotic bactericidal assays

Antibacterial assays were performed as previously described (Peng et al., 2015b). Briefly, overnight bacteria were collected by centrifugation for 5 min at 8,000 rpm. Then washed with 0.85% sterile saline three times, and diluted to an OD_600_ of 0.2 in 5 mL of M9 minimal medium (containing 10 mM acetate, 1 mM MgSO_4_, and 100 µM CaCl_2_). Relevant metabolites and/or antibiotics were added to the samples, which were then incubated at 37°C for 6 h. Finally, 100 µL of the culture was obtained and then serially diluted. An aliquot (10 µL) of each dilution was plated on LB agar to determine the bacterial count.

### Metabolomics and data analysis

Sample preparation was performed as previously described (Cheng et al., 2014). In brief, 10 mL OD_600_ of 1.0 cells were incubated in M9 minimal medium for 6 h, with or without uracil. Equivalent amounts of cells were collected by centrifugation at 8,000 rpm for 5 min, quenched using liquid nitrogen, and cold methanol was added prior to storage at −80°C. Cells were lysed by sonication for 5 min at a 200 W power setting, and metabolites were extracted with 1 mL of cold methanol (Sigma, United States) containing 10 μg of ribitol (Sigma, United States, used as the internal standard). Samples were centrifuged at 12,000 *g* at 4°C for 10 min and the supernatant was dried using a rotary vacuum centrifuge (LABCONCO, Germany). The dried extracts were then incubated with 50 µL of methoxyamine hydrochloride solution (20 mg/mL in pyridine) at 37°C for 1.5 h. Subsequently, samples were derivatized *via* a 37°C reaction for 1 h with the addition of 50 µL of N-methyl-N-(trimethylsilyl) trifluoroacetamide. Finally, the derivatized sample was centrifuged at 4°C and 12,000 *g* for 15 min, and the supernatant was removed for analysis. 1 µL of the derivatized sample was injected and analyzed using the gas chromatography–mass spectrometry (GC-MS-QP 2010 plus, Shimadzu, Japan). The inlet temperature, split ratio of the carrier gas (high-purity helium), and constant linear velocity were set at 300 °C, 5:1, and 40.0 cm/s respectively (Ye G. Z. et al., 2021). The metabolites were separated using the DB-5 MS capillary column (30 m × 250 μm × 0.25 μm, J&W Scientific Inc., Unite States). Mass signals of metabolites were acquired in the full scan mode. To obtain the retention index of the bacterial metabolites, which is equivalent to the retention time of n-alkanes, a light diesel sample was analyzed using the same instrumental parameters as those of the analytical samples.

Raw mass spectrometry data in NetCDF format exported by GC-MS solution 4.2 (Shimadzu, Japan) was used to perform peak processing using the XCMS method (Smith et al., 2006). Metabolites were firstly identified by searching the commercial mass spectra libraries, and then verified using available standards. Significance analysis was conducted using IBM SPSS Statistics 22.0 (SPSS Inc., Chicago, IL, United States), and statistically significant results were considered if the *p*-value was less than 0.05. R studio software version 4.0.3 was employed for hierarchical cluster analysis (HCA), and SIMCA-P + software 12.0 (Umetrics, Umeå, Sweden) was used for principal component analysis (PCA) and orthogonal partial least squares-discriminant analysis (OPLS-DA). Z-score analysis was performed based on standard deviations from the mean using Microsoft Excel, and the enrichment pathways of significant metabolic was performed with MetaboAnalyst 5.0. Interactive pathways (iPath) analysis was conducted using iPath 3.0 (https://pathways.embl.de/). Data was processed using Microsoft Excel and figures were generated using GraphPad Prism 8.0 (San Diego, CA, United States).

### Quantitative RT-PCR analysis

Quantitative real-time PCR (qRT-PCR) was carried out as described previously, with modifications (Chen et al., 2022). Bacterial cells (2 mL) were harvested at OD_600_ = 1.0 by centrifugation (12,000 g, 4°C, 3 min) and immediately quenched in liquid nitrogen. Then, the cells were lysed to extract total RNA using the bacterial RNA extraction kit (Vazyme, Jiangsu, China). Reverse transcription-PCR was conducted with 1 µg of total RNA, using the *Evo M-mLV* RT Mix Kit with gDNA Clean for qPCR (Accurate Biotechnology Co., Ltd., Guangdong, China), according to the instruction manual. The qRT-PCR was carried out in 96-well plates, each well containing a total volume of 20 µL liquid-composed of 10 µL 2× SYBR Green Pro Taq HS Premix, 9 µL PCR-grade water, 0.2 µL cDNA template, and 0.4 µL each of the primers (10 μM). Primers sequences are listed in Supplementary Table S1. As per the manufacturer’s instructions, three biological replicates were used, and all assays were performed on the CFX Connect Real-Time System (Bio-Rad, USA). Cycling parameters were as follows: an initial denaturation at 95°C for 30 s, 45 cycles at 95°C for 5 s, and 58°C for 30 s mRNA levels of target genes were normalized to that of *gyrB*, which is constitutively and steadily expressed under the conditions analyzed (Hartmann et al., 2014).

### Measurement of enzyme activity

Enzymatic activity was measured as previously described (Chen et al., 2022). In brief, cells were collected and then resuspended in sterile saline to OD_600_ = 1.0 in M9 medium after washing three times. Samples (30 mL) were collected by centrifugation at 8,000 rpm for 5 min. Cells were resuspended following lysis using lysostaphin and sonication (200 W total power with 60% output, 2 s pulse, and 3 s pause) over ice for 20 min. Enzymatic activity was determined spectrophotometrically by monitoring the reduction of 3-(4,5-dimethyl-2-thiazolyl)-2,5-diphenyl-2H-tetrazolium bromide (MTT) at 566 nm using the following reaction mixtures. The samples were centrifuged at 12,000 *g* for 10 min and concentration of protein concentration was determined using a BCA assay (Beyotime, Shanghai, China). Supernatants containing 200 µg of total proteins were added to an α-ketoglutaric dehydrogenase (OGDH) reaction mixture containing 0.15 mM MTT, 2.5 mM MgCl_2_, 5 mM phenazine methosulfate (PMS), 0.2 mM thiamine pyrophosphate (TPP), 5 mM α-ketoglutaric acid potassium salt, and 100 mM PBS [pH 7.2]. An isocitrate dehydrogenase (ICDH) reaction mixture (0.15 mM MTT, 1 mM PMS, 2.5 mM MgCl_2_, 100 mM Tris–HCl [pH 8.8], 3.5 mM isocitrate) with 10 µg of total proteins from supernatants was added to a succinate dehydrogenase (SDH) reaction mix (0.15 mM MTT, 1 mM PMS, 2 mM sodium succinate and 100 mM PBS), making up a final volume of 200 µL in a 96-well plate. Subsequently, the plate was incubated at 37°C for 20 min in the dark and absorbance value by colorimetric readings was measured at 566 nm. The activity of citrate synthase (CS) and complex I of electron transport chain was measured using the citrate synthase assay kit (Suzhou Keming Biotechnology Co., Ltd., Suzhou, China) and complex I activity assay kit (Beijing Solarbio Science and Technology Co., Ltd., Beijing, China).

### Measurement of ATP and NAD^+^/NADH

ATP levels were determined *via* the BacTiter-GloTM Microbial Cell Viability Assay (Promega, Madison, WI, United States), as previously described (Ye J. Z. et al., 2021). Overnight bacterial cultures were collected by centrifugation at 8,000 rpm for 5 min. Samples were washed three times with 0.85% sterile saline, suspended in M9 minimal medium, and diluted to the concentration with an OD_600_ of 0.2. Uracil (0, 1.25, 2.5, 5, or 10 mM) was added to the medium, and incubated at 37°C and 220 rpm for 6 h. After 10-fold dilution, 50 µL of the sample and 50 µL of the kit solution were added to a 96-well plate together. Finally, the absorbance was measured in the microplate reader (Biotek, Synergy HT, Vermont, USA) according to the manufacturer’s instructions.

The NAD^+^/NADH assay kit with WST-8 (Beyotime, Shanghai, China) was used to measure NAD^+^/NADH. First, cells were collected and diluted to an OD_600_ value of 1.0 with or without uracil (10 mM), in M9 minimal media. After 6 h of incubation, 5 mL of cell pellets were washed with PBS (pH = 7.2) and resuspended in 600 µL of liquid mixture (precooled extraction buffer: PBS, 1:1). Subsequently, pellets were lysed with lyases by sonication for 20 min, and then centrifuged at 12,000 *g* for 10 min at 4°C. Absorbance of the supernatants was measured at 450 nm using a plate reader (Biotek, Synergy HT, Vermont, USA) with a reference wavelength of 655 nm.

### Measurement of ROS and bacterial respiration

Overnight bacterial cultures were pelleted by centrifugation at 8,000 rpm for 5 min. Samples were washed three times with 0.85% sterile saline, suspended in M9 minimal medium, and diluted to an OD_600_ of 0.6. Uracil (10 mM) or/and gentamicin (400 μg/mL) were added to the medium and incubated at 37 °C, 220 rpm for 6 h. Then, 194 µL of bacterial cells and 4 µL of 2′,7′-dichlorodihydrofluorescein diacetate (DCFH-DA, Sigma, United States), which of the final concentration was 20 µM, and added to a 96-well plate and incubated at 37°C for 1 h in the dark. Fluorescence units were immediately measured at an excitation wavelength of 485 nm and emission wavelength of 515 nm using a plate reader (Biotek, Synergy HT, Vermont, Unites States).

The role of uracil in bacterial respiration was determined based on the changes in iodonitrotetrazolium chloride (INT) (Sigma, USA) (Liu Y. et al., 2021). Briefly, overnight bacterial cultures were washed, and diluted to an OD_600_ value of 1.0 in M9 minimal media, with or without uracil (10 mM). Subsequently, 1 mM INT and 0.6 mM NADH (Macklin Biotech Co., Ltd., Shanghai, China) were added as substrates: the solution was then incubated for 45 min in the dark at 37°C. The reaction was terminated by addition of 5% trichloroacetic acid. Insoluble formazan was collected by centrifugation at 13,000 *g* for 5 min and then extracted with ethanol. Absorbance of the supernatant was measured at 485 nm every 5 min for 45 min.

### Measurement of membrane potential

DiOC_2_3) was used to measure membrane potential (PMF) (Novo et al., 1999). In brief, overnight bacteria were collected at an OD_600_ value of 0.2, and incubated in 5 mL of M9 minimal medium for 6 h at 37°C and 220 rpm. Cells (495 μL) containing 1 × 10^7^ CFU were stained with 5 μL of 3 mM DiOC_2_3) for 30 min in the dark. Red (PE) and green (FTIC) fluorescence intensity was determined using a flow cytometer (Beckman Coulter, Brea, CA, United States). The PMF was calculated with formula 1.5 + log10 (red/green).

### Measurement of intracellular gentamicin concentration

A gentamicin ELISA rapid test kit (CUSABIO, Hubei, China) was used to detect intracellular gentamicin content. In brief, overnight bacteria were collected at a concentration with an OD_600_ value of 1.0. Subsequently, gentamicin was added and incubated for 6 h at 37°C and 220 rpm, with or without uracil. 30 mL of cells were harvested, lysated with lysostaphin (Macklin Biotech Co., Ltd., Shanghai, China) and sonication (200 W total power with 60% output, 2 s pulse, and 3 s pause) over ice for 20 min. The supernatant was collected after centrifugation at 12,000 *g* for 10 min at 4°C. Finally, gentamicin concentration was assessed based as per the rapid diagnostic kit instructions.

### Bacterial culture and infection of mouse

Bacterial cultures from −80°C were diluted 1: 100 in fresh 50 mL LB medium, and cultured to an OD_600_ of 1.0 at 37°C, and oscillated at 220 rpm. Following collection by the centrifugation at 8,000 rpm for 5 min, cells were diluted with PBS to the concentration with 5 × 10^9^ CFU/mL cells. BALB/c mice (8–10 weeks) were injected with 100 μL of the bacterial suspensions with the concentration of 5 × 10^9^ CFU/mL cells. Bacterial infection was performed by tail vein injection. After 6 h, mice were randomly divided into four groups (n = 8 per group), and separately treated with PBS, gentamicin (5 mg kg^−1^), uracil (50 mg kg^−1^), and gentamicin (5 mg kg^−1^) + uracil (50 mg kg^−1^) twice a day. The survival of mice was recorded daily for 7 days.

## Results

### Exogenous uracil promotes aminoglycosides to kill gram-positive resistant bacteria

**FIGURE 1:**
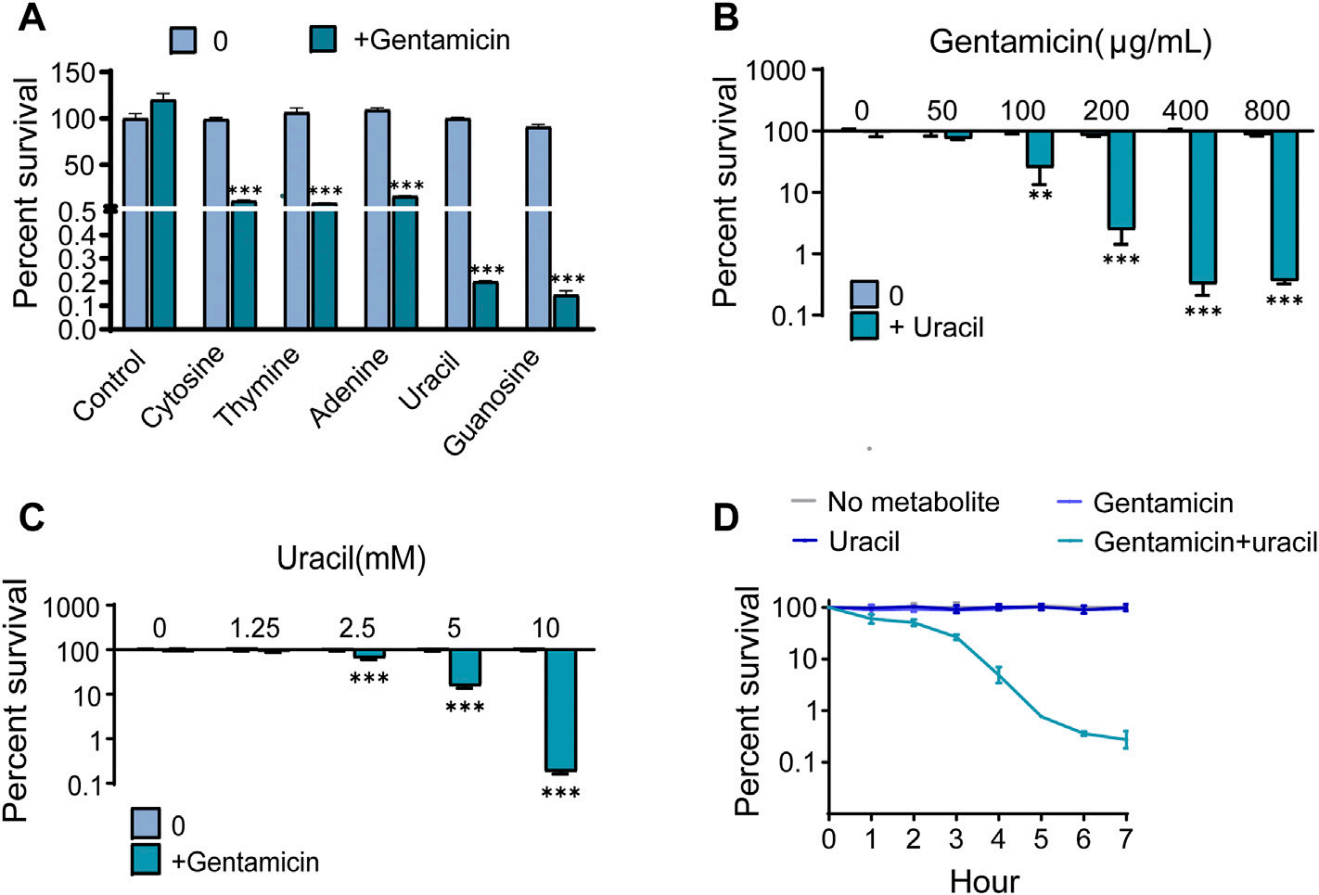
Uracil promotes gentamicin killing against Gram-positive bacteria USA300. **(A)** Percent survival of USA300 when incubated with different nucleotides. **(B)** Percent survival of USA300 when incubated with different concentrations of uracil plus 400 μg/mL gentamicin. **(C)** Percent survival of USA300 when incubated with different concentrations of gentamicin plus 10 mM uracil. **(D)** The time effect of combined bactericidal efficacy of treatment with and without 10 mM uracil or/and 400 μg/mL gentamicin. All data are displayed as mean ± SEM. *p < 0.05, **p < 0.01, ***p < 0.001, determined by one-way ANOVA.

To observe the effect of nucleotides on antibiotic activity against methicillin-resistant *S. aureus*, USA300, was incubated with gentamicin or gentamicin plus five single exogenous nucleotides. More than 95% of the cells survived when exposed to 400 μg/mL gentamicin alone. However, the bactericidal effect of gentamicin was significantly increased by combined treatment with cytosine, thymine, adenine, uracil or guanine (Figure 1A). In particular, the efficacy of gentamicin was potentiated approximately 400-fold by uracil or guanine. Due to the strong synergistic bactericidal effect of uracil and its wide application in therapy, we further explored the role of uracil in synergistic sterilization with aminoglycosides (Ramesh et al., 2020). We found that exogenous uracil increased gentamicin-mediated bacterial killing in a dose-dependent manner (Figure 1B). In addition, the synergistic efficacy of gentamicin with uracil was enhanced with an increase in the gentamicin dose and time (Figures 1C, D). Similar synergistic bactericidal effects of uracil in combination with other aminoglycosides (amikacin, kanamycin, and tobramycin) were also observed (Supplementary Figure S1A). However, uracil did not augment the bactericidal effect of other types of antibiotics (ampicillin, tetracycline, erythromycin, and ciprofloxacin) against USA300 (Supplementary Figure S1B). To determine whether the enhanced-bactericidal effects of uracil and gentamicin in combination persisted until extended periods of time, viable bacteria were killed repeatedly for 20 days (1 cycle/day). The synergistic bactericidal effect on the 10th day was 10 folds weaker than that on the first day, but after 20 days, the efficacy was restored (Supplementary Figure S1C). These results indicate that uracil can effectively and consistently potentiate aminoglycosides activity to eliminate MRSA.

### Uracil induces significant changes in the metabolic profile of bacteria

**FIGURE 2:**
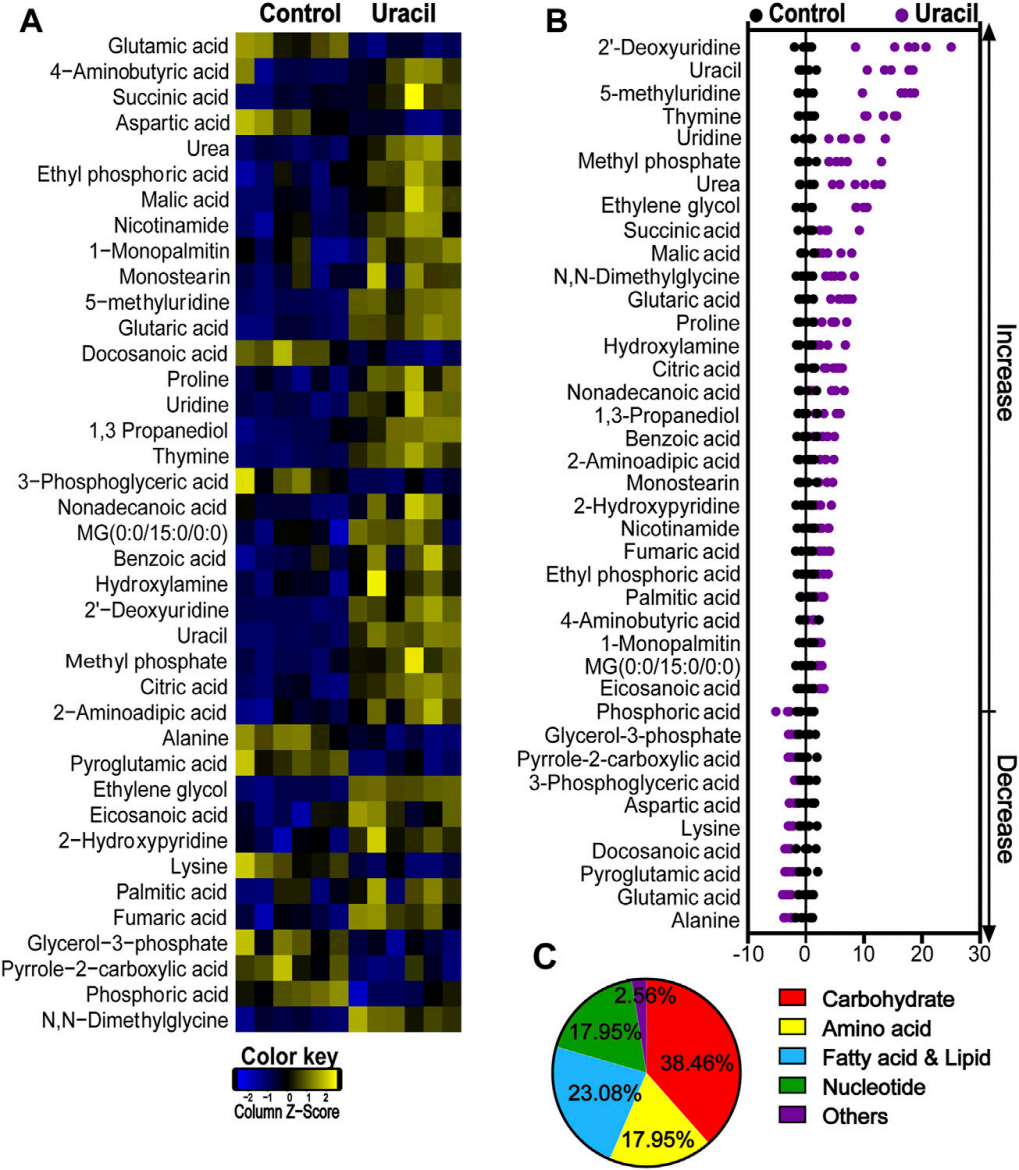
Changes in differential metabolites of USA300 in response to uracil treatment. **(A)** Heat map showing differential abundance of metabolites. Blue and yellow colors indicate lower and higher abundances of the metabolites relative to the mean level of the control group, respectively (see color scale). **(B)** Z-score plots of changes in differential metabolites based on control. The data were respectively scaled to the mean and standard deviation of control. Each point represents one biological repeat. Different treatments are distinguished by the color. **(C)** Categories of differential abundance of different metabolites.

To explore whether uracil played a role through metabolic regulation, which potentiated gentamicin-mediated killing. GC-MS was used to analyze metabolomes of USA300 cells in the presence or absence of uracil. Unsupervised hierarchical clustering identified that 65 metabolites in the bacteria (Supplementary Figure S2A). Among these metabolites, 32.31%, 20%, 29.23%, 13.85%, and 4.61% of metabolites were carbohydrates, amino acids, fatty acids and lipids, nucleotides, and others (Supplementary Figure S2B). In total, 39 metabolites showed differential abundances (*p* < 0.05) with uracil (Figure 2A). The Z-score plot demonstrated that upon uracil treatment, 2′-deoxyridine, uracil, 5-methyluridine, thymine and uridine were the top five most upregulated metabolites, while alanine, glutamic acid, pyroglutamic acid, docosanoic acid and lysine were the top five most downregulated metabolites (Figure 2B). These differential metabolites were mainly divided into five categories: carbohydrates (38.46%), amino acids (17.95%), fatty acids (23.08%), nucleotides (17.95%), and others (2.56%) (Figure 2C). These results indicate that exogenous uracil induces significant reprogramming of the MRSA metabolome.

### Exogenous uracil promotes the TCA cycle

**FIGURE 3:**
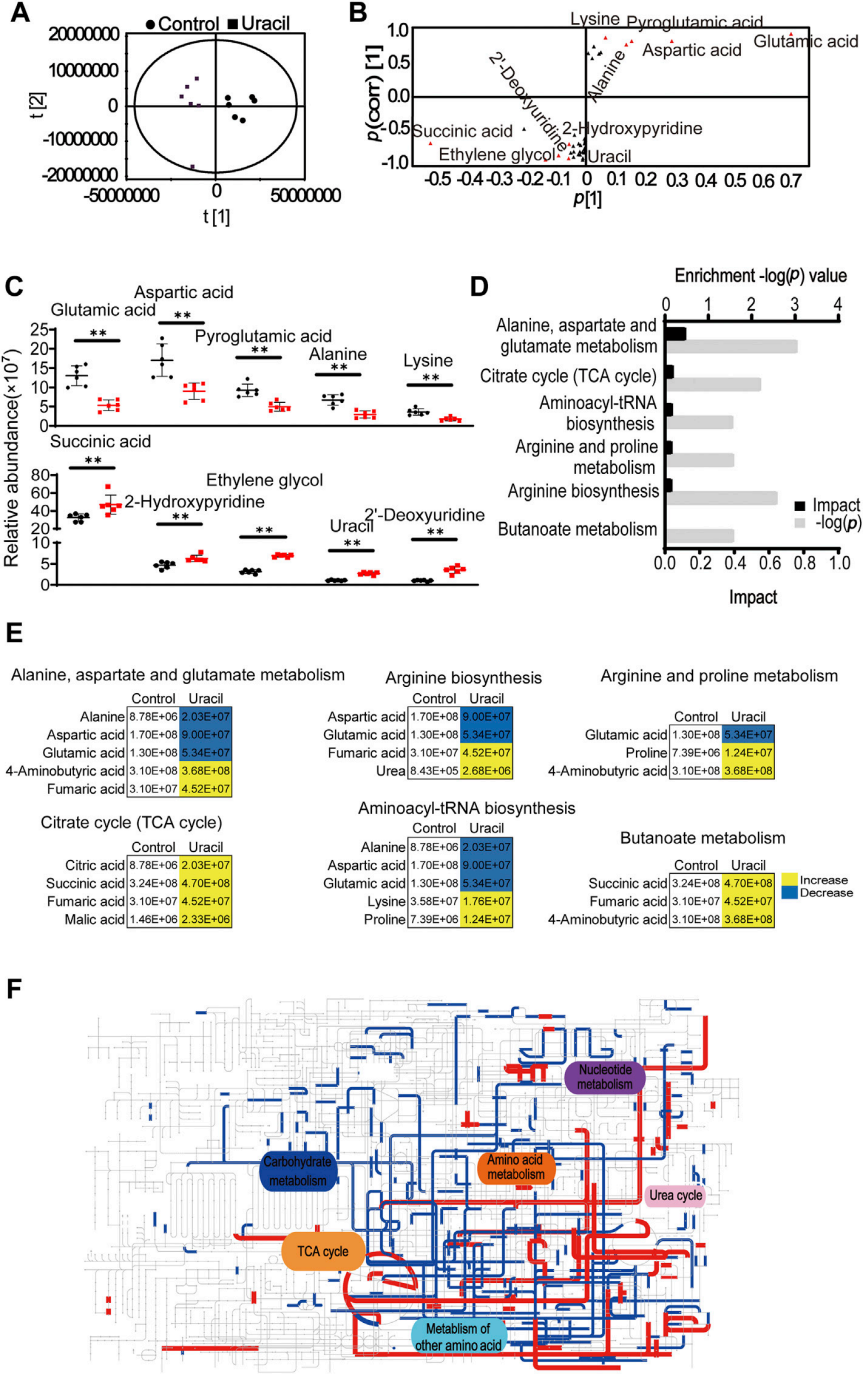
Enrichment of metabolic pathways in USA300 in response to uracil treatment. **(A)** The score plot of PCA. Each dot represents one biological replica in the plot. **(B)** S-plot of OPLS-DA. Triangle represents individual metabolite. Red indicates the potential biomarkers, which is greater than or equal to 0.05 and 0.5 for absolute value of covariance *p* [1] and correlation *p* (corr) [1], respectively. **(C)** The scatter plot of biomarkers in data. **(D)** Significantly enriched metabolic pathways in response to uracil treatment (*p* < 0.05). **(E)** Changes in differential metabolites involved in the significantly enriched pathways. Yellow color and blue color indicate increased and decreased metabolites, respectively, in uracil-treated group. **(F)** IPath integrated analysis of changes in the metabolic pathways. Red and blue lines represent increased and decreased metabolisms, respectively, in uracil-treated group. Totally, 39 significant metabolites (*p* < 0.05) were used for the analysis (https://pathways.embl.de).

Principal component analysis was used to identify two principal components, where component t [1] distinguished USA300 from USA300 + uracil (Figure 3A). The discriminating variables are presented in an S-plot (Figure 3B). Cutoff values for the absolute value of the covariance *p* and correlation *p* (corr) were ≥0.05. The axes that are plotted in the S-plot from the predictive component are *p* vs. *p* (corr), representing the magnitude (modeled covariation) and reliability (modeled correlation), respectively. The selection of potentially biochemically interesting compounds needs a combination of covariance and correlation information, which is the purpose of the S-plot (Wiklund et al., 2008). Ten crucial biomarkers were identified, of which alanine, lysine, aspartic acid, glutamic acid, and pyroglutamic acid had decreased. In contrast, succinic acid, ethylene glycol, uracil, 2′-deoxyuridine, and 2-hydroxy pyridine increased in response to uracil treatment (Figure 3C). Increase in succinic acid, an intermediate of the TCA cycle, as well as the elevation of aspartic acid and glutamic acid, metabolites capable of directly entering the TCA cycle–suggest a potential role for the TCA pathway. Consistently, results of the pathway enrichment analysis showed that six metabolic pathways were significantly enriched, of which alanine, aspartate, and glutamate metabolism, TCA cycle, and aminoacyl-tRNA biosynthesis exhibited highest impact (Figure 3D). Bacterial metabolites enriched in the TCA cycle and butanoate metabolism had all increased in response to uracil treatment, including citric acid, succinic acid, fumaric acid, malic acid, and 4-aminobutyric acid (Figure 3E). Further analysis using iPath 3.0 provided a global overview of the metabolome, revealing increased TCA cycle and nucleotide metabolism, and decreased carbohydrate metabolism in USA300 upon treatment with uracil (Figure 3F). These results demonstrate that uracil promotes the TCA cycle in MRSA.

### Promotion of the TCA cycle is responsible for uracil-enabled killing

**FIGURE 4:**
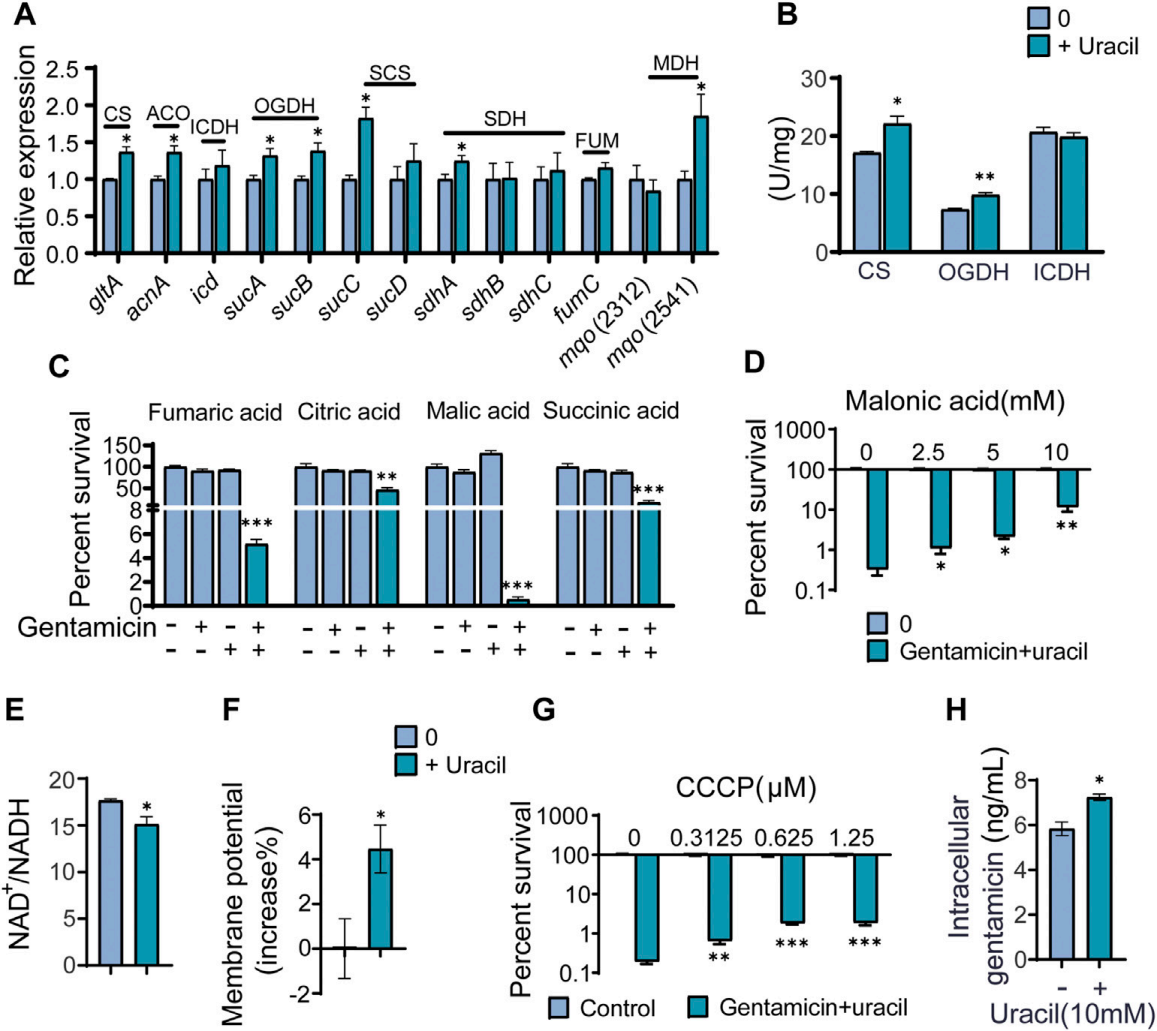
The mechanism of exogenous uracil promotes metabolic flux of USA300. **(A)** Changes in mRNA expression levels of genes involved in the TCA cycle. **(B)** Activity of citrate synthase (CS), α-ketoglutarate dehydrogenase (OGDH) and isocitrate dehydrogenase (ICDH) were detected with and without 10 mM uracil. **(C)** Percent survival of USA300 when incubated at 400 μg/mL gentamicin with four intermediates (the concentrations of fumaric acid, citric acid, malic acid, and succinic acid was 5 mM) in the TCA cycle. **(D)** The concentration effect of malonic acid on the bactericidal efficacy of treatment with 400 μg/mL gentamicin plus 10 mM uracil. **(E)** Uracil affected the ratio of NAD^+^/NADH. **(F)** The membrane potential of USA300 with and without uracil. **(G)** The concentration effect of CCCP on the bactericidal efficacy of treatment with 400 μg/mL gentamicin plus 10 mM uracil. **(H)** Intracellular gentamicin content was detected in presence of uracil and gentamicin. All data are displayed as mean ± SEM. *p < 0.05, **p < 0.01, ***p < 0.001, determined by one-way ANOVA.

To further validate that the TCA cycle was enhanced upon uracil treatment, expression levels of genes and the activity of enzymes involved in the TCA cycle were measured. Among the 14 genes detected, mRNA expression levels of seven genes were elevated, including *glta*, *acnA*, *sucA/B/C*, *sdhA*, and *mqo* (2,541), while the remainder were unchanged in the presence of uracil (Figure 4A). Genes exhibiting elevated expression encoded for enzymes including one subunit of citrate synthase (CS), aconitate hydratase (ACO), succinyl-CoA synthetase (SCS), succinate dehydrogenase (SDH), malate dehydrogenase (MDH), and two subunits of α-ketoglutarate dehydrogenase (OGDH) in the TCA cycle. To further confirm the activation of the TCA cycle, the activity of three key enzymes in the TCA cycle, CS, isocitrate dehydrogenase (ICDH), and OGDH were measured. Consistent with qRT-PCR results, the activity of CS and OGDH were increased in USA300 cells following uracil treatment (Figure 4B).

Since metabolomics showed that citric acid, succinic acid, fumaric acid, and malic acid were increased in USA300 exposed to exogenous uracil, these four metabolites were separately added to the medium to investigate their roles in enhancing aminoglycoside activity. We found that the aforementioned metabolites could independently promote bactericidal effects of gentamicin on USA300 (Figure 4C). Blocking the TCA cycle with malonic acid (a competitive inhibitor of SDH) rescued the viability of the bacteria exposed to uracil, demonstrating the crucial role of the TCA cycle in uracil-mediated synergistic sterilization with aminoglycosides (Figure 4D). Hence, we speculated that uracil might affect NADH and PMF. As expected, the intracellular NAD^+^/NADH ratio decreased and PMF increased after exogenous uracil treatment (Figures 4E, F). Subsequently, PMF inhibitor carbonyl cyanide-chlorophenyl hydrazone (CCCP) was added to the medium to examine the role of PMF in uracil-mediated synergistic sterilization with aminoglycosides. The results showed that uracil-enabled killing of USA300 by gentamicin was decreased following CCCP treatment (Figure 4G). Uniformly, exogenous uracil increased intracellular gentamicin content in USA300 cells (Figure 4H). Taken together, uracil treatment elevates TCA cycle flux and promotes the conversion of NAD^+^ to NADH and increases PMF, thereby enhancing gentamicin uptake and potentiating its bactericidal effects.

### Uracil enhances bacterial respiration and ROS generation

**FIGURE 5:**
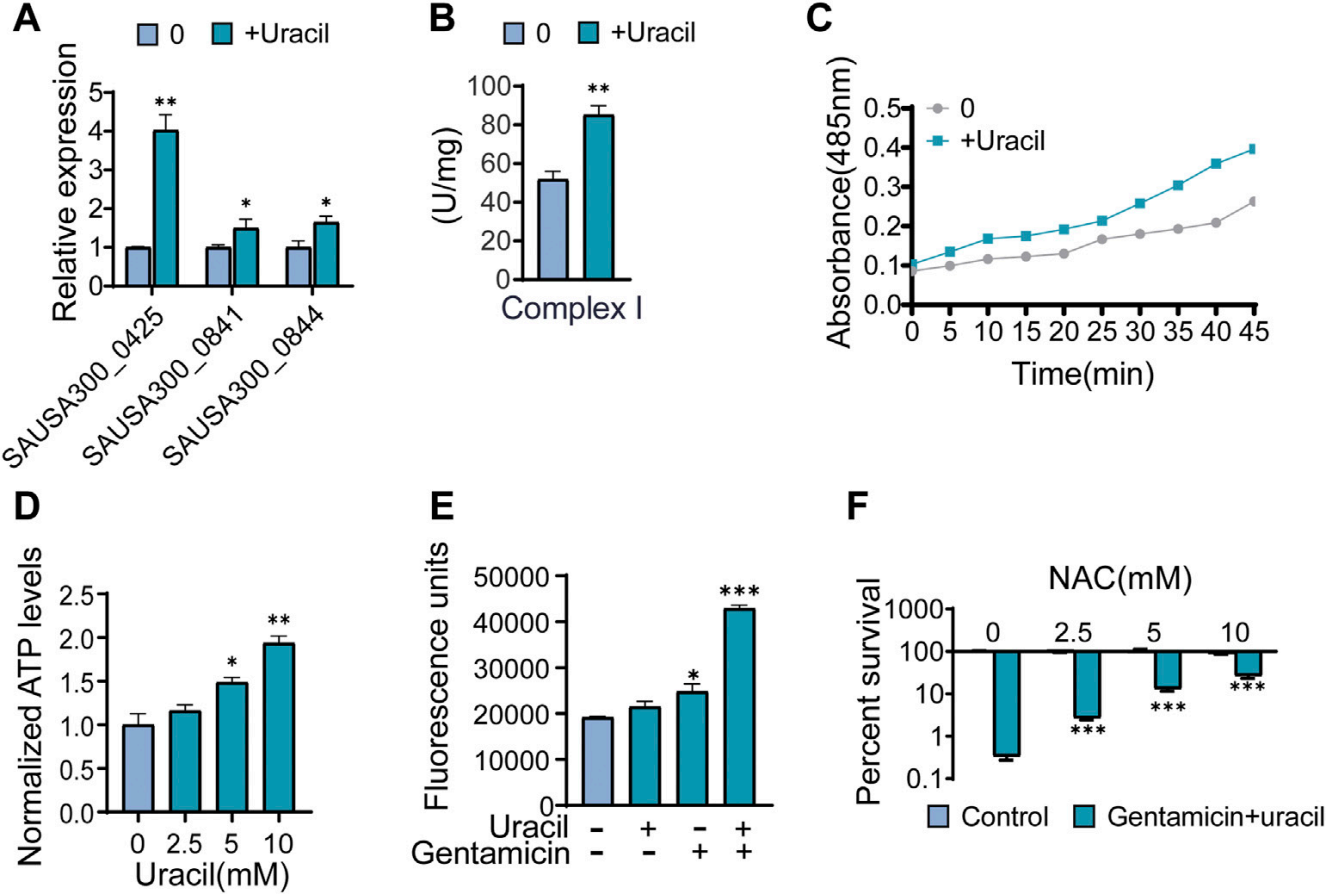
Uracil sensitizes Gram-positive USA300 to gentamicin killing through ROS. **(A)** The gene expression levels of complex I including SAUSA300-0425 (NADH dehydrogenase I, F subunit), SAUSA300-0841 (conserved hypothetical protein), and SAUSA300-0844 (conserved hypothetical protein) in presence of uracil. **(B)** The activity of complex I in presence of uracil. **(C)** Change of bacterial respiration in presence of uracil. **(D)** Change of ATP levels in response to uracil. **(E)** Gentamicin and/or uracil affected the production of ROS. RFU, relative fluorescence units. **(F)** Percent survival of USA300 when incubated at 400 μg/mL gentamicin and 10 mM uracil with different concentrations of N-acetyl-L-cysteine (NAC). All data are displayed as mean ± SEM. *p < 0.05, **p < 0.01, ***p < 0.001, determined by one-way ANOVA.

As the TCA cycle plays a key role in aerobic respiration, uracil may affect the bacterial electron transport chain. Thus far, it has been shown that two types of respiratory oxygen reductases are present; however, bc1 complex and cytochrome c oxidase do not exist in *S. aureus* (Schurig-Briccio et al., 2014). Thus, the activity and gene expression of complex I in the respiration chain were determined. As expected, the activity and gene expression of complex I were increased in uracil-treated USA300 (Figures 5A, B). Meanwhile, uracil increased bacterial respiration in a time-dependent manner (Figure 5C). In addition, uracil promoted the production of the intracellular ATP level in USA300 (Figure 5D). Increased bacterial respiration is typically accompanied by the production of reactive oxygen species (ROS). We observed that uracil alone increased the production of ROS, and this increase was higher following combined treatment with gentamicin and uracil (Figure 5E). Consistently, adding the ROS scavenger N-acetyl-L-cysteine (NAC) blocked the synergistic bactericidal activity of gentamicin plus uracil, demonstrating the involvement of ROS in uracil-mediated synergistic sterilization with aminoglycosides (Figure 5F). These results indicate that exogenous uracil promotes bacterial respiration and elevates the ROS levels, thereby increasing the efficacy of aminoglycosides.

### Uracil improves gentamicin efficacy on pathogenic bacteria *in vitro* and *in vivo*

**FIGURE 6:**
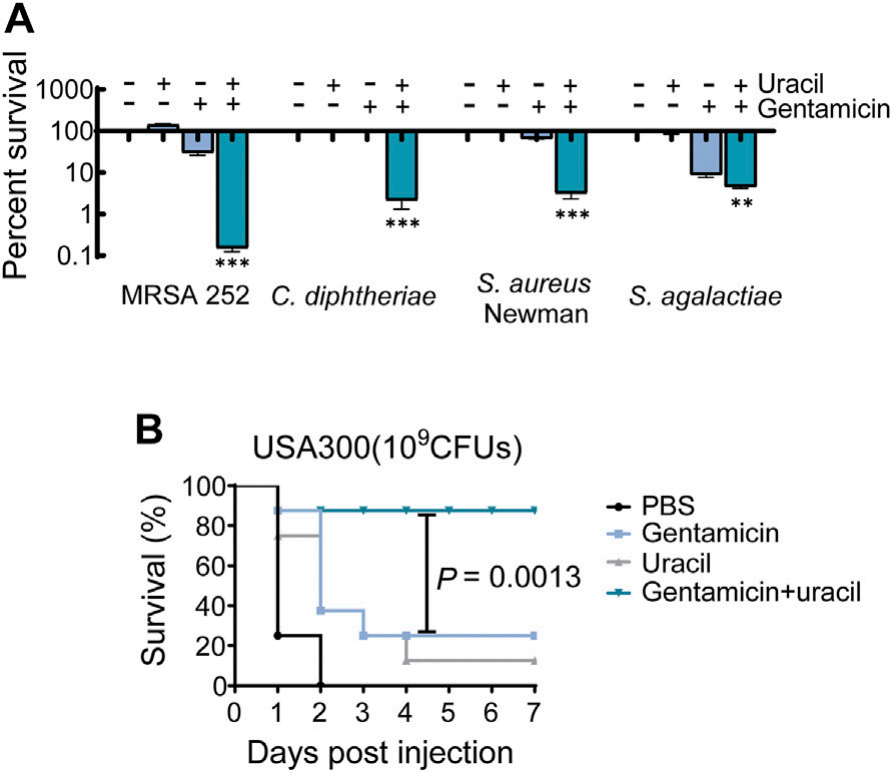
Uracil improves gentamicin killing against pathogenic bacteria and survival of mice. **(A)** Killing effects of treatment with uracil (10 mM) plus gentamicin killing against MRSA252, *C. diphtheriae*. *S. aureus* Newman and *S. agalactiae*, which concentration of gentamicin was 400 μg/mL, 0.8 μg/mL, 800 μg/mL and 4 μg/mL, respectively. **(B)** Percent survival of mice infected by the *S. aureus*, USA300. After 6 h post-infection, then treated by PBS, gentamicin (5  mg kg^−1^), uracil (50 mg kg^−1^), and both. Survival of mice were monitored during 7 days. All data are displayed as mean ± SEM. *p < 0.05, **p < 0.01, ***p < 0.001, determined by one-way ANOVA.

The synergistic bactericidal effects of uracil and gentamicin against other pathogenic bacteria were further examined *in vitro*. The results showed that uracil increased the efficacy of gentamicin (Figure 6A). A mouse model of MRSA infection was used to assess the synergistic effect *in vivo*. BALB/c mice were infected with USA300 and treated with a single dose of gentamicin and/or uracil. Gentamicin or uracil treatment in isolation were ineffective in improving the survival of infected mice (only 10% for uracil and 20% for gentamicin) (Figure 6B). Interestingly, the combined administration of gentamicin and uracil greatly increased the survival of infected mice (>80%) (Figure 6B). These data suggest that uracil increases the killing efficacy of gentamicin against other Gram-positive bacteria *in vitro*. Moreover, combined treatment significantly improve survival rate in mice infected with MRSA.

## Discussion

MRSA is regarded as a major threat to global healthcare and is resistant to most antibiotics, resulting in high morbidity and mortality (Liu W. T. et al., 2021; Nandhini et al., 2022). Therefore, it is necessary to explore the mechanisms underlying antibiotic resistance and to seek solutions. In addition to advances in genomics, metabolomics has recently been utilized to study the mechanisms underlying antibiotic resistance. The metabolic status of bacteria was found to play a crucial role in mediating resistance (Zhang et al., 2019; Chen et al., 2022; Kok et al., 2022; Zheng et al., 2022). Moreover, exogenous metabolites including carbohydrates, amino acids, and nucleotides were found to modulate antibiotics susceptibility and bactericidal effects of antibacterial drugs by metabolic reprogramming in Gram-negative bacteria (Peng et al., 2015a; Peng et al., 2015b; Su et al., 2015; Wu et al., 2015; Ye et al., 2018; Wang et al., 2020; Chung et al., 2022). Metabolites present several advantages as adjuvants for antibiotics as they are inexpensive, non-toxic, and slow to induce resistance. Uracil is an exogenous metabolite widely used in cancer treatment (Ramesh et al., 2020) and also exhibits synergistic activity with antibiotics to treat Gram-negative bacteria (Yang et al., 2019). However, there is currently no clear and systematic evidence elucidating the contribution of nucleotides to the bactericidal effects of antimicrobial drugs, especially against Gram-positive bacteria.

In the present study, we discovered that uracil effectively increase gentamicin to eliminate MRSA USA300 approximately 400-fold. As far as we know, this is the best synergistic effect with ineffective gentamicin against MRSA. Subsequently, using GC-MS-based metabolomics, we found that upon uracil treatment, the TCA cycle was activated and four intermediates in the pathway were increased in USA300 cells. Moreover, intermediates in the pathway were shown to promote gentamicin-mediated killing. A previous study found that a dysfunctional TCA cycle reduced the susceptibility of *S. epidermidis* to β-lactam antibiotics (Chittezham Thomas et al., 2013). Consistent with this notion, blocking TCA cycle with malonic acid, a competitive inhibitor of succinate dehydrogenase, could rescued USA300 viability upon treatment with uracil and gentamicin. We also found that the transcription of seven genes and activity of two key enzymes in the TCA cycle were elevated, while the NAD^+^/NADH ratio decreased, thereby increasing PMF in USA300 upon treatment with uracil. In the electron transport chain, NADH is the driving force for PMF production, which plays an important role in the bacterial uptake of aminoglycosides (Taber et al., 1987; Allison et al., 2011). Confirming this, an uncoupler of bacterial PMF (CCCP) (Lewis et al., 1994), also weakened the synergistic action of gentamicin and uracil in USA300. We also demonstrated that the increased uptake of aminoglycosides by enhancing PMF contributes to the bactericial activity of aminoglycosides.

Bacterial respiration, ROS, and ATP levels were also measured in this study. Upon treatment with uracil, we found higher bacterial respiration, as evidenced by increased ATP and ROS levels, and increased activity of complex I in the respiratory chain in USA300. Uracil alone could enhance bacterial respiration and result in an increase in ROS levels, however, this increase may not be lethal. Notably, the ROS level was significantly higher in USA300 cells treated with gentamicin plus uracil compared to those treated in isolation. NAC, a ROS scavenger (Liu et al., 2020b), was found to weaken the bactericidal activity of combined treatment with gentamicin and uracil. The inhibitory effect of NAC might be related to decreased basal respiration induced by metabolic alterations, thereby minimizing metabolic toxicity and antibiotic lethality (Lopatkin et al., 2021). Accordingly, we found that ROS mediated the bactericidal effect suggesting that increased uptake of aminoglycosides may contribute to the generation of lethal ROS (Kohanski et al., 2007; Hong et al., 2017; Van Acker and Coenye, 2017).

## Conclusion

**FIGURE 7:**
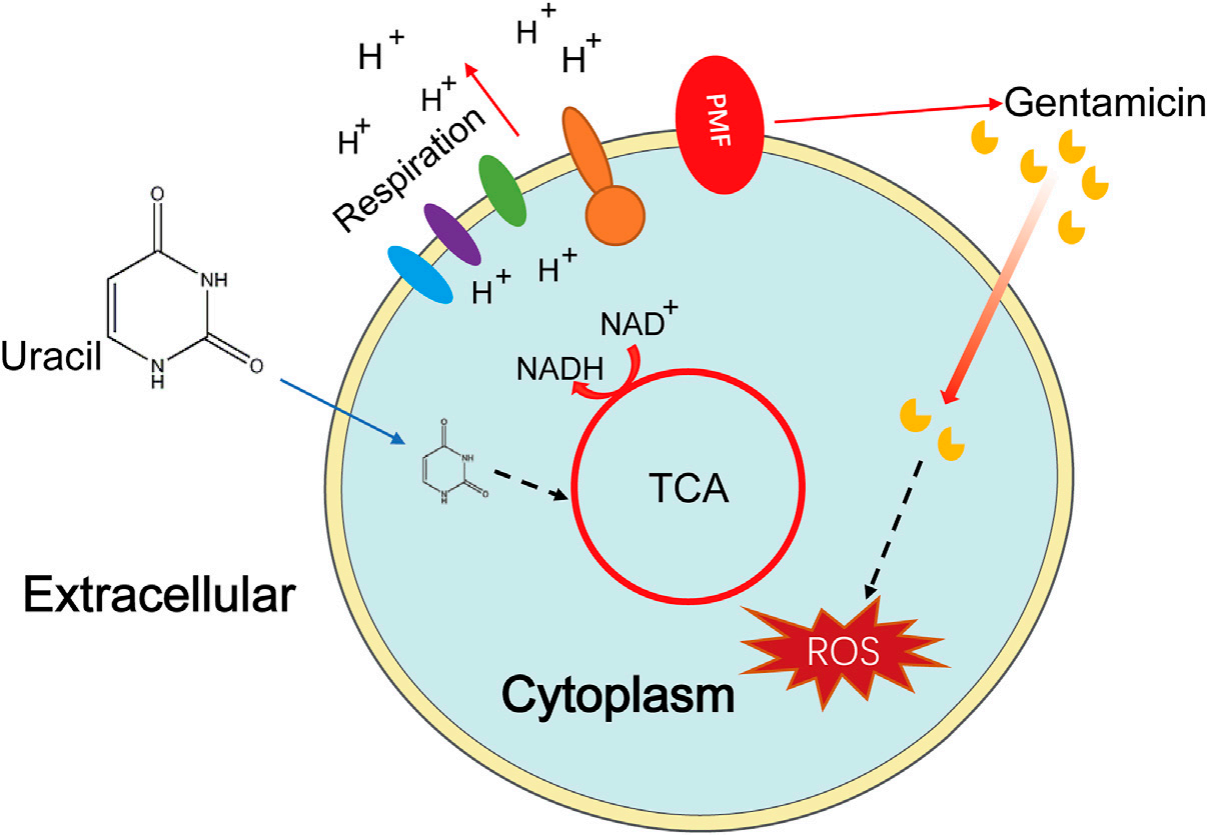
Sketch diagram describes a killing mechanism of uracil and gentamicin in USA300. TCA cycle is enhanced by uracil resulting in a series of effects, including increasing PMF and stronger respiration, thus uptaking of more gentamicin and increasing production of ROS, leading ultimately to bacterial cell death.

In conclusion, our results demonstrate that uracil reprograms the metabolism of MRSA, especially increasing the TCA cycle and cellular respiration, leading to increased uptake of aminoglycosides and ROS generation (Figure 7). Although uracil improved the survival rate of MRSA-infected mice, several additional studies are required prior to practical clinical applications. This study presents a novel metabolite-based strategy to reprogram bacterial metabolism, then improve the efficacy of aminoglycosides against MRSA and other pathogens.

## Data Availability

The datasets presented in this study can be found in online repositories. The names of the repository/repositories and accession number(s) can be found below: https://www.ncbi.nlm.nih.gov; OQ179915.1, OQ186749.1, OQ179937.1, OQ179932.1 and OQ186750.1; MRSA251.
