# Supplementary material for: Uracil restores susceptibility of methicillin-resistant Staphylococcus aureus to aminoglycosides through metabolic reprogramming
Source: Front Pharmacol. 2023 Jan 24;14:1133685. doi: 10.3389/fphar.2023.1133685 (PMC9902350; doi:10.3389/fphar.2023.1133685)
Supplement: Supplementary file 1 [file Table1.DOCX]

**Supplementary Table 1**

| Primers for qRT-PCR | | | |
| --- | --- | --- | --- |
| Gene | Primer (5'-3') | Gene | Primer (5'-3') |
| *gltA-*F | GTGCGGTATCATCATTGTCA | NADH dehydrogenase I, F subunit-F (*SAUSA300_0425*) | GCATGGCACGTCTTGTTG |
| *gltA-*R | CCGAAGCCCATTACTTTATC | NADH dehydrogenase I, F subunit-R (*SAUSA300_0425*) | TCACTGTAGGCGGCGTTGT |
| *acnA-*F | TTGCTGATGAGGACCTATTA | conserved hypothetical protein-F (*SAUSA300_0841*) | AAGCAATGGCTAAATGAACC |
| *acnA-*R | CTTCACCGTCATTACCTTTAC | conserved hypothetical protein-R (*SAUSA300_0841*) | AAATAGAAGCGAGTCGTCCT |
| *icd-*F | TCGGTGATGGAATTGGACC | conserved hypothetical protein-F (*SAUSA300_0844*) | TAGTGAAACATTCGGCATCG |
| *icd-*R | TGGCCAGCTAGCACTTCTT | conserved hypothetical protein-R (*SAUSA300_0844*) | GCACCACCAACTAAGATAGAT |
| *sucA-*F | TCTGAACAAATGCGACCAT | *sdhB-*F | GTGCACAAGCAATCTCGC |
| *sucA-*R | TGTAACCTTATCCGCTTGA | *sdhB-*R | ACACTGCTGTAAGCCACCA |
| *sucB-*F | GCCAGAGGTTAAAGTTCCA | *fumc-*F | TGTATGGCAAACAGGAAGC |
| *sucB-*R | CACCTGCTTCTTCAGATACAA | *fumc-*R | ACATCATCATTTGGGTGGA |
| *sucC-*F | TGCTGCGAAGACTCCTGAA | *mqo* (2312)*-*F | TGATTACAGCAGGGCAAC |
| *sucC-*R | ATGGGTTGATTTCTACGATTGA | *mqo* (2312)*-*R | TAACCAAATGACGGCACCA |
| *sucD-*F | GTTGTAGGCTTTATCGGTGG | *mqo* (2541)*-*F | GGTATCCCTGAAAGTAAACATT |
| *sucD-*R | CTGAAGGTGTTGCCGCTG | *mqo* (2541)*-*R | GAGGTACAGTCATTGGTGGTG |
| *sdhA-*F | TGGTGCGGTCAATACTAAA | *gyrB-*F | CCAGGTAAATTAGCCGATTGC |
| *sdhA-*R | TACGCCCATACGGTCTAAT | *gyrB-*R | AAATCGCCTGCGTTCTAGAG |
| *sdhC-*F | CCATTTATGGCAAACACGT |  |  |
| *sdhC-*R | TTGAAGTCCACCCCAAGT |  |  |
