# Supplementary figures and images for: Uracil restores susceptibility of methicillin-resistant Staphylococcus aureus to aminoglycosides through metabolic reprogramming
Source: Front Pharmacol. 2023 Jan 24;14:1133685. doi: 10.3389/fphar.2023.1133685 (PMC9902350; doi:10.3389/fphar.2023.1133685)

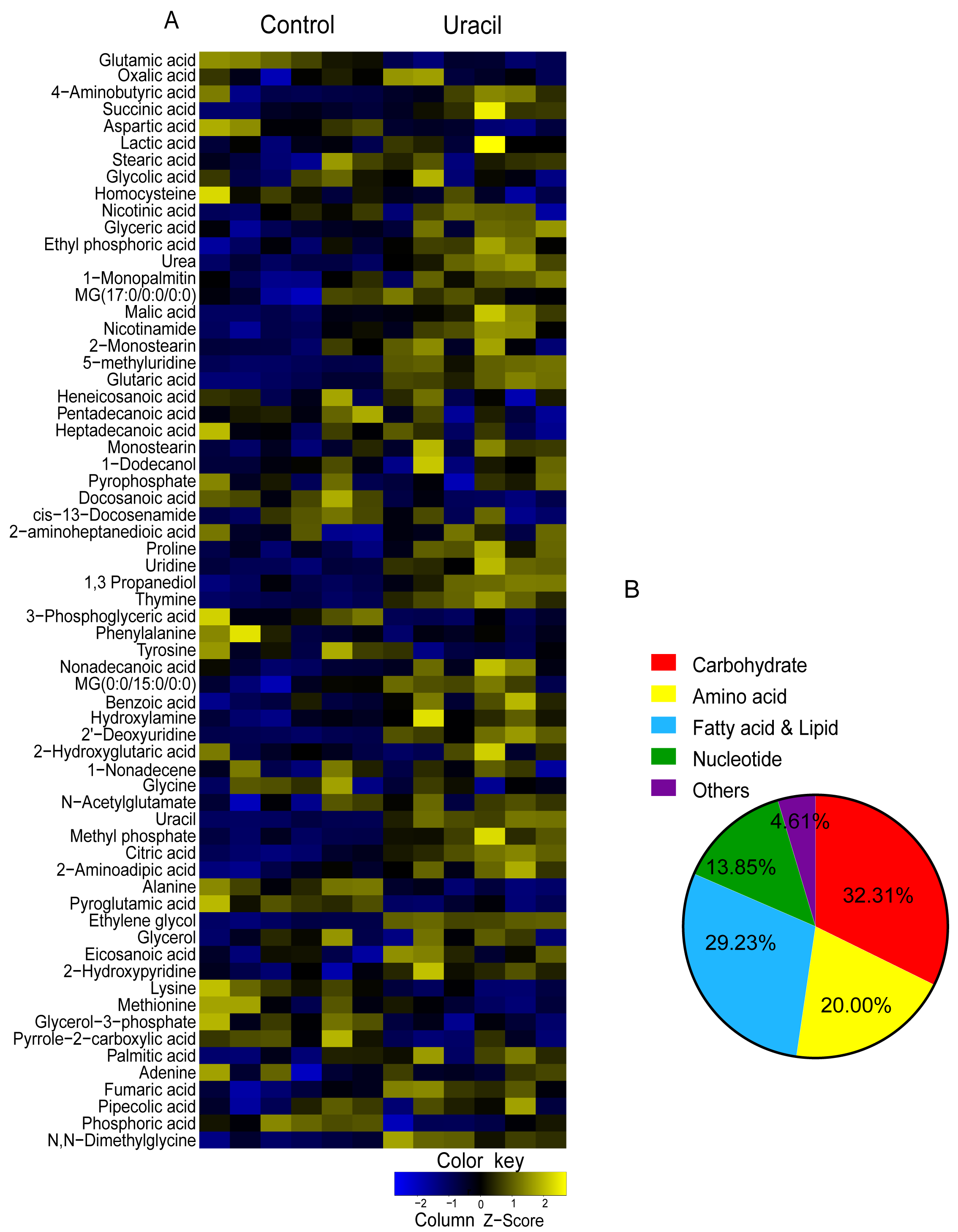

Supplement: Supplementary file 2 [file Image2.TIF]

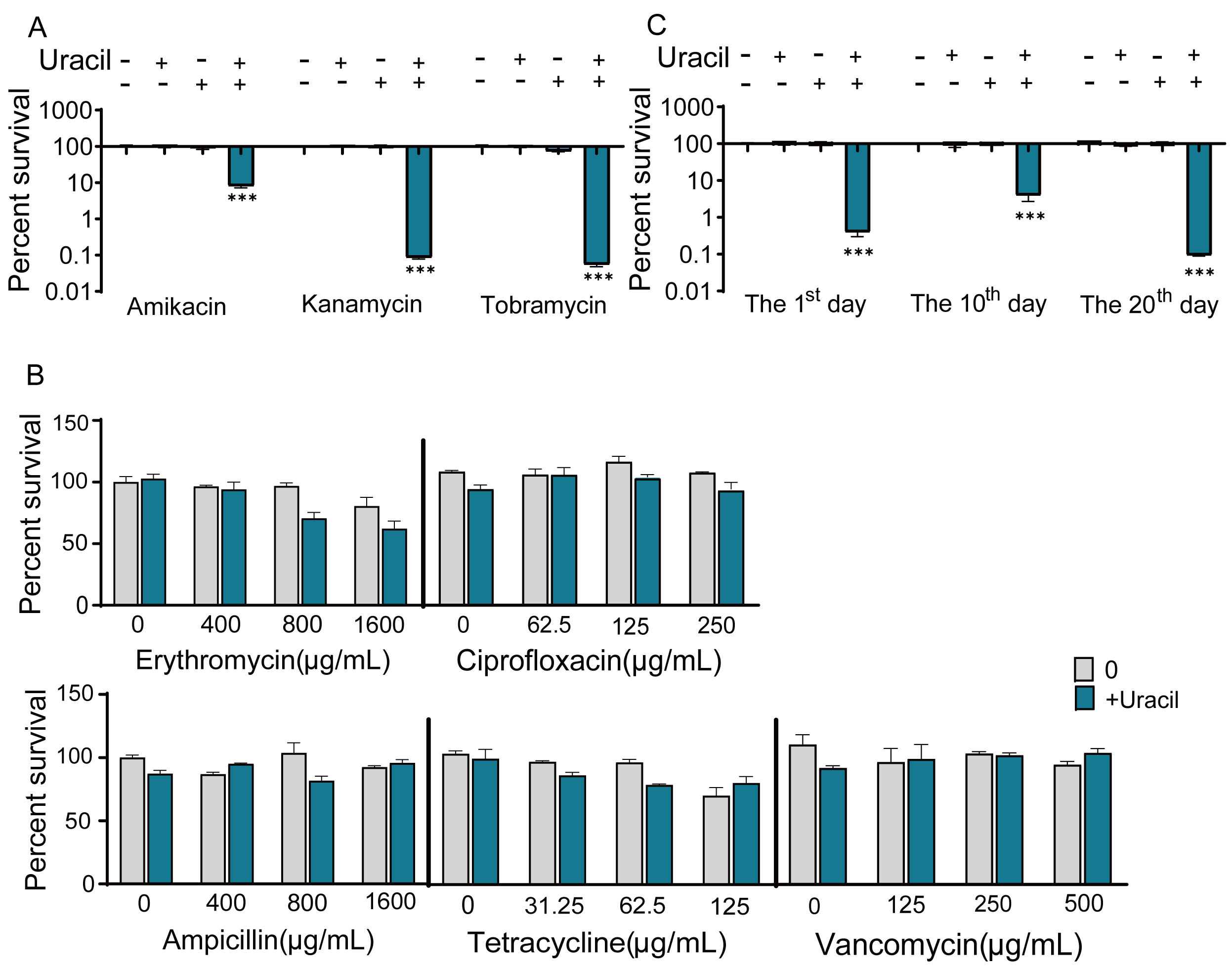

Supplement: Supplementary file 3 [file Image1.TIF]
